# Genome-Wide Identification of Apple Ubiquitin SINA E3 Ligase and Functional Characterization of MdSINA2

**DOI:** 10.3389/fpls.2020.01109

**Published:** 2020-07-24

**Authors:** Hong-Liang Li, Xun Wang, Xing-Long Ji, Zhi-Wen Qiao, Chun-Xiang You, Yu-Jin Hao

**Affiliations:** State Key Laboratory of Crop Biology, College of Horticulture Science and Engineering, Shandong Agricultural University, Tai’an, China

**Keywords:** apple, bioinformatics analysis, expression analysis, *MdSINA2*, ABA sensitivity

## Abstract

SINA (Seven in absentia) proteins are a small family of ubiquitin ligases that play important roles in regulating plant growth and developmental processes as well as in responses to diverse types of biotic and abiotic stress. However, the characteristics of the apple SINA family have not been previously studied. Here, we identified 11 *MdSINAs* members in the apple genome based on their conserved, N‐terminal RING and C-terminal SINA domains. We also reconstructed a phylogeny of these genes; characterized their chromosomal location, structure, and motifs; and identified two major groups of *MdSINA* genes. Subsequent qRT-PCR analyses were used to characterize the expression of *MdSINA* genes in various tissues and organs, and levels of expression were highest in leaves. *MdSINAs* were significantly induced under ABA and carbon- and nitrate-starvation treatment. Except for MdSINA1 and MdSINA7, the other MdSINA proteins could interact with each other. Moreover, MdSINA2 was found to be localized in the nucleus using *Agrobacterium*-mediated transient expression. Western-blot analysis showed that MdSINA2 accumulated extensively under light, decreased under darkness, and became insensitive to light when the RING domain was disrupted. Finally, ABA-hypersensitive phenotypes were confirmed by transgenic calli and the ectopic expression of *MdSINA2* in *Arabidopsis*. In conclusion, our results suggest that *MdSINA* genes participate in the responses to different types of stress, and that *MdSINA2* might act as a negative regulator in the ABA stress response.

## Introduction

Ubiquitination is one of the most important post-translational modifications in plant development and the responses of plants to environment (Devoto et al., 2003; Xu and Jaffrey, 2013; Stone, 2014; Zhang et al., 2015). The ubiquitin-proteasome system consists of ubiquitin (Ub), ubiquitin activating enzyme (E1), ubiquitin-binding enzyme (E2), ubiquitin ligase (E3), and the 26S proteasome, and functions in the degradation of target proteins in a three-enzyme cascade manner (Sadanandom et al., 2012; Jansen et al., 2014). Ubiquitin molecules are small spherical (76 residues) and highly conserved proteins that can covalently modify target proteins to alter their stability, function, and localization (Schimke, 1973; Callis et al., 1995; Dametto et al., 2015). Initially, E1 activates Ub to form the E1-Ub complex with ATP. The activated Ub is transferred to the cysteine residue of the E2 ubiquitin-binding enzyme, forming an intermediary. The E3 ubiquitin ligase then recognizes the E2-Ub intermediary and the substrate protein, and transfers the Ub from the E2-Ub intermediary to the target protein. Finally, the substrate protein labeled by ubiquitin can be transported to the 26S proteasome for degradation (Santner and Estelle, 2010).

Ubiquitin ligases bind to specific ubiquitin-binding enzymes and target proteins, and the E3 ligase determines the diversity and specificity of the target protein. In the plant kingdom, ubiquitin ligase can be classified into three types based on its reaction mechanism and special conserved domains: HECT (Homology to E6-Associated Carboxy-Terminus) ubiquitin ligases, RING (Really Interesting New Gene) ubiquitin ligases, and U-box ubiquitin ligases (Duplan and Rivas, 2014). Among ubiquitin ligases, RING type E3 ligases have been the most extensively studied. RING type E3 ligases contain a conserved RING domain that consists of histidine and cysteine and can bind to zinc ions (Stone et al., 2005; Metzger et al., 2014). RING ubiquitin ligases can be further divided into two groups: single subunit RING ubiquitin ligases and multi-subunit RING ubiquitin ligases. Multi-subunit RING ubiquitin ligases can be subdivided into four types: Skp1-Cullin-F-box, VHL-ELONGIN-CUL2/5, BTB-CUL3 and DDB-CUL4 (Hua and Vierstra, 2011; Sadanandom et al., 2012). In recent years, RING finger ubiquitin E3 ligases have been extensively studied and have been shown to play important roles in plant growth and development as well as in response to stress.

SINA (seven in absentia) proteins, RING-finger E3 ligases that were first identified in *Drosophila* (Carthew and Rubin, 1990), are involved in regulating the differentiation of light receptors (Li et al., 1997). Most *SINA* family members possess a highly conserved N-terminal RING finger domain and a C-terminal SINA domain. The N-terminal domain is for binding to E2, and the SINA domain recognizes the target protein which is subsequently degraded by the 26S proteasome (Hu and Fearon, 1999; Den Herder et al., 2012). In addition, studies have found that SINA homologs can regulate their target proteins to adapt to different developmental stages and environmental changes. For example, SINAT5^Ler^ (Landsberg ecotype) can mediate the degradation of the transcriptional activator NAC1 which is involved in the auxin signaling pathway, thereby regulating lateral root development in *Arabidopsis* (Xie et al., 2002). Further study has shown that SINAT5^Ler^ could interact with FLC (Flowering Locus C), LHY (LATE ELONGATEDHYPOCOTYL), and DET1 (DE-ETIOLATED1) to regulate flowering time in *Arabidopsis* by promoting the degradation of FLC and LHY (Park et al., 2007; Park et al., 2010). SINAT2 is involved in carotenogenesis by interacting with RAP2.2 (Welsch et al., 2007). All SINATs can interact with dephosphorylated BES1, which is one of the core transcription factors involved in BR signaling. However, only SINAT5^Ler^ is known to be able to negatively regulate BR signaling by mediating the degradation of BES1.

Other major findings include the accumulation of SINATs proteins in light and their degradation in the dark (Yang et al., 2017) as well as the diverse synergistic and antagonistic functions of SINA members. For example, SINAT1 and SINAT2 can negatively regulate starvation-induced autophagy by ubiquitinating ATG6 (AUTOPHAGY PROTEIN6) or ATG13 (AUTOPHAGY PROTEIN13). Conversely, SINAT6 promotes autophagy by increasing the number of autophagic puncta under nutrient-rich or nutrient-poor conditions (Qi et al., 2017; Qi et al., 2020). A recent study has demonstrated that the ectopic expression of tomato *SlSINA4* resulted in cell death in *N. benthamiana* leaves, but overexpression of any of the other five *SlSINAs* can suppress the hypersensitive response and cell death (Wang et al., 2018). Abscisic acid (ABA) plays a critical role in plant growth and stress adaptation (Knight and Knight, 2001). Overexpression of *SINA2* (SEVEN IN ABSENTIA 2) increases tolerance to drought by inducing the closure of stomata in *Arabidopsis* (Bao et al., 2014). OsDIS1, a homologous protein of SINA in rice, is a negative regulator in the drought response (Ning et al., 2011). In addition, Siah1 and Siah2, human SINA homologs are involved in multiple processes such as synaptic transmission, apoptosis, tumor suppression, and stress response (Wheeler et al., 2002; Franck et al., 2006; Khurana et al., 2006; Fukuba et al., 2007).

Our knowledge of the SINA family in apple is limited. Here, we identified 11 *MdSINA* members in apple using bioinformatics analyses. We analyzed tissue expression patterns and the responses of these genes to abiotic stress. Additionally, we assessed the ability of these genes to form homodimers and heterodimers using yeast two-hybrid (Y2H) assays. Our results provide basic information on the function of SINA proteins in apple.

## Materials and Methods

### Identification of *SINA* Gene Family Members in Apple

Five SINA homologous proteins in *Arabidopsis thaliana* were obtained from TAIR (https://www.arabidopsis.org) (Lamesch et al., 2012). All protein sequences in apple (*Malus* × *domestica*) were downloaded from Apple Genome and Epigenome (https://iris.angers.inra.fr/gddh13/the-apple-genome- downloads.html) (Daccord et al., 2017). Local BLAST with an E-value of 1×10^-5^ was used to screen *MdSINA* genes. We confirmed the blast result by SMART (http://smart.embl-heidelberg.de/) and analyzed molecular weights and theoretical pI by ProtParam (http://web.expasy.org/protparam/). Subcellular localizations of MdSINAs were predicted by WoLF PSORT II (https://www.genscript.com/wolf-psort.html) (Horton et al., 2007).

### Multiple Sequence Alignment and Phylogenetic Analysis

Clustal Omega (https://www.ebi.ac.uk/Tools/msa/clustalo/), an online server, was used to perform multi-sequence alignment between the amino acid sequences of apple and *A. thaliana*, and Jalview (Jalview 2.10.5) was used to edit and visualize the comparison results.

MEGA: X was used to construct a neighbor-joining evolutionary tree with 1,000 bootstrap replications and a Poisson model (Kumar et al., 2018). Six SINA proteins in rice (*Oryza sativa)* were obtained from the Phytozome database (https://phytozome.jgi.doe.gov/pz/portal.html) (Goodstein et al., 2012). In MEGA X, ClustalW method was selected for sequence alignment, partial deletion and 95% site coverage cutoff were used gaps/missing data treatment.

### Gene Structure, Chromosomal Location, and Conserved Motif Analysis

We used GSDS 2.0 (http://gsds.cbi.pku.edu.cn/) and MG2C (http://mg2c.iask.in/mg2c_v2.0/) to display the structure and chromosomal locations of *MdSINA* genes (Hu et al., 2015). Genome annotation information (gene_models_20170612.gff3.bz2) was downloaded from Apple Genome and Epigenome (https://iris.angers.inra.fr/gddh13/the-apple-genome-downloads.html). Conserved motifs were detected by MEME (http://memesuite.org/tools/meme). WebLogo (http://weblogo.berkeley.edu/logo.cgi) was used to redraw the motifs (Crooks et al., 2004; Bailey et al., 2009).

### Synteny Analysis

The syntenic blocks information of the apple genome can be downloaded from the Plant Genome Duplication Database (http://chibba.agtec.uga.edu/duplication/). The genome sequence of the homologous SINA proteins in apples was evaluated using BLASTP. The MCScanX algorithm (Wang et al., 2012) was used to scan syntenic blocks containing the apple SINA genes.

### Plant Materials and Treatments

Materials included various apple tissues, tissue-cultured apple seedlings (*Malus × domestic* “Royal Gala”), “Orin” apple calli, “Columbia” (WT) *Arabidopsis* seedlings (Col-0), and tobacco (*Nicotiana benthamiana*). The growth conditions of tissue-cultured apple seedlings, “Orin” apple calli, *Arabidopsis* seedlings and tobacco were the same as those described previously (An et al., 2019). Three-week-old shoot tissue culture seedlings were used for rooting with 0.2 mg/L IAA. We collected the roots, stems, leaves, flowers, and fruits of a 7-year-old “Gala” apple tree, which were immediately frozen in liquid nitrogen and stored at 80°C for tissue expression analysis.

For ABA treatment, 3-week-old apple seedlings were treated with 100 μM ABA for 0, 1, 3, 6, 12, and 24 h following previously described methods (Zhao et al., 2019) and were then immediately frozen in liquid nitrogen and stored at −80°C for subsequent analysis. For the carbon-deprivation treatment, 3-week-old apple seedlings with roots grown in MS medium were treated without sucrose and in the dark for 0, 6, 12, 24, 36, and 48 h. For nitrate-deprivation treatment, 3-week-old seedlings grown on MS medium were transferred to nitrogen-deficient MS liquid medium and grown under normal growth conditions for 0, 6, 12, 24, 36, and 48 h.

### RNA Extraction and qRT-PCR Analysis

Total RNAs of apple samples and calli was isolated using a RNA extraction kit (Tiangen, Beijing, China) and used for qRT-PCR analysis following previously described methods (Ren et al., 2019). Three technical and biological replicates were performed to detect the transcripts of *MdSINA* genes. Specific primers of the *MdSINA* genes were designed and used for qRT-PCR analysis (**Supplementary Table 1**).

### Vector Construction and Genetic Transformation

The open reading frames of *MdSINA2* was cloned into the pRI-GFP vector to construct the overexpression vector. The primer pairs *MdSINA2*-F (5′-GTCGAC ATGGACTTGGAAAGCATCGAGTGT-3′)/−R and (5′-GGATCC GCTACACAGGTTTGGTATGCA-3′) were used to amplify the full-length *MdSINA2*. “Orin” apple calli were infected by the recombinant plasmids *35S::GFP-MdSINA2* and WT (*35S::GFP*) *via* the *Agrobacterium*-mediated transformation method as previously described (An et al., 2016). The transgenic *Arabidopsis* were obtained by using the floral dip transformation method (Clough and Bent, 1998).

### Yeast Two‐Hybrid (Y2H) Assays

Interactions among MdSINA proteins were characterized by Y2H assays. The full-length cDNA of *MdSINA*s was cloned and inserted into yeast vector pGBT9 and pGAD424. The recombinant plasmids of pGAD424-MdSINAs and pGBT9-MdSINAs were transformed into yeast “Y2H Gold.” The yeast was grown on selection medium as described previously (An et al., 2018); the primer pairs used are listed in **Supplementary Table 2**.

### Subcellular Localization of MdSINA2

The recombinant plasmid *35S::GFP-MdSINA2* and the control vector *35S::GFP* were injected into the epidermal cells of *N. benthamiana* leaves *via Agrobacterium*-mediated transient expression. The fluorescent signals were observed by a laser confocal microscope (Zeiss LSM 510 META, Jena, Germany).

### Protein Extraction and Western-Blotting

The total protein of 2-week-old *MdSINA2-GFP* transgenic calli was isolated as described (Li et al., 2012). After the western blot assay, anti-GFP (Sigma-Aldrich) was used to detect protein abundance. Actin served as a protein loading control.

### ABA-Sensitive Assays

Transgenic and WT calli were cultivated on MS medium containing 100 μM ABA. Fresh weight and superoxide anions content were measured after 18 days. The superoxide anions generation rates were determined by spectrophotometry using their corresponding assay kits (Keming, Suzhou, China).

Homozygous seeds of WT and transgenic *Arabidopsis* were plated on MS medium with or without either 1 or 3μM ABA and grown at 22°C under an 8/16 h dark/light photoperiod. Photographs were taken after 8 days.

### Statistical Analysis

All experiments were performed in triplicate. Error bars showed the standard deviation of three biological replicates. GRAPHPAD PRISM 6.01 software was used to conduct a one-way ANOVA to detect significant differences (*, P < 0.05).

## Results

### Genome-Wide Identification of Apple *SINA* Genes

Five SINATs were previously identified in the* A. thaliana* genome, including AtSINA1 (SINAT1), AtSINA2 (SINAT2), AtSINA3 (SINAT3), AtSINA4 (SINAT4), and AtSINA5 (SINAT5^Ler^) (Xie et al., 2002; Wang et al., 2008). Five SINAT protein sequences were used to blastp in the apple genome database. After software screening and manual detection based on the conserved domains, 11 MdSINAs were obtained (**Table 1**). The *MdSINA *genes were named based on their chromosomal locations (*MdSINA1–11*). We found that the 11 *MdSINA* genes were unevenly distributed on eight chromosomes in the apple genome. Gene distribution density was highest on chromosome 7 (three genes), followed by chromosome 1 (two genes). The range of lengths of the MdSINA proteins was small, from 287 (MdSINA10/11) to 334 (MdSINA8) amino acids, and the molecular weights were predicted to be between 32,548–37,952 Da. The theoretical isoelectric point ranged from 6.49 (MdSINA7) to 8.55 (MdSINA5). In addition, the subcellular locations of the 11 *MdSINA* genes were predicted, suggesting that the MdSINA proteins were localized in either the cytoplasm or nucleus (**Table 1**).

**Table 1:** Basic information of *SINA* family members in apple.

Gene Name	Gene ID	Chromosome Localization of Gene	Number of Amino Acids	Molecular Weight	Theoretical pI	Subcellular Localization
MdSINA1	MD01G1082000	Chr01:18851198…18855278	319	36208.29	6.62	Cyto
MdSINA2	MD01G1218700	Chr01:31083173…31086177	333	37952.97	6.55	Nucl
MdSINA3	MD02G1290000	Chr02:34598424…34602594	305	34800.85	7.88	Nucl
MdSINA4	MD03G1012000	Chr03:919096…923194	308	34847.85	8.1	Cyto
MdSINA5	MD06G1109400	Chr06:24892232…24897122	309	35453.74	8.55	Nucl
MdSINA6	MD07G1037000	Chr07:3041621…3045379	305	34689.57	6.88	Nucl
MdSINA7	MD07G1150400	Chr07:21874143…21877048	315	35655.61	6.49	Nucl
MdSINA8	MD07G1288600	Chr07:34966306…34969128	334	38005.99	6.55	Nucl
MdSINA9	MD11G1014900	Chr11:1183310…1187186	308	34771.88	7.82	Cyto
MdSINA10	MD12G1055100	Chr12:6229125…6233025	287	32721.58	8.09	Cyto
MdSINA11	MD14G1054300	Chr14:5470040…5474302	287	32548.37	7.85	Cyto

### Phylogenetic and Synteny Analyses of Apple *SINA* Genes

**Figure 1:**
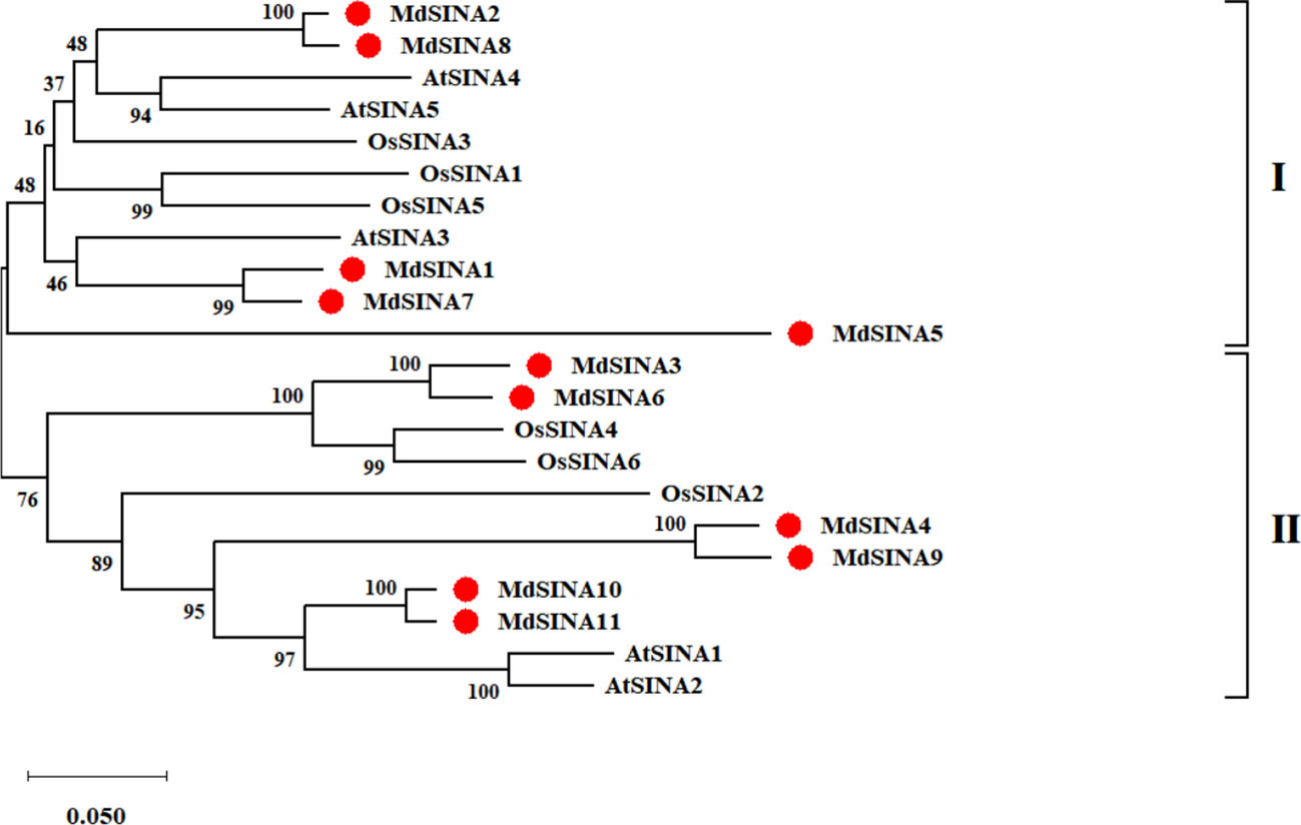
Phylogenetic analysis of *SINA* genes in apple, *Arabidopsis*, and rice. The phylogenetic tree was constructed using the NJ method and a bootstrap test with 1000 replicates.

**Figure 2:**
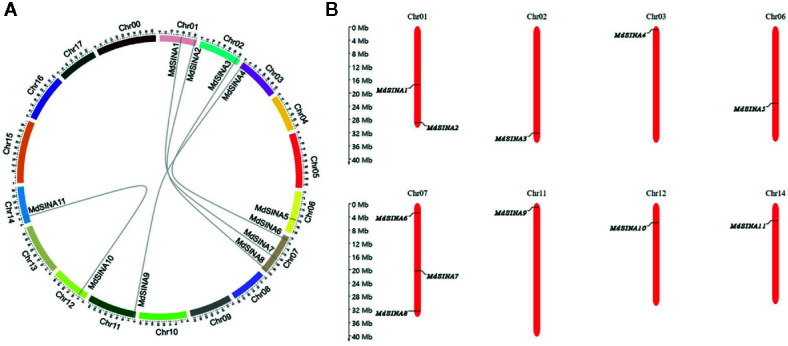
Evolutionary relationships among *MdSINA* gene family members and the chromosomal distribution of *SINA* genes. **(A)** Synteny analysis of *SINA* genes in apple. **(B)** Chromosomal location of *SINA* genes in apple.

We performed a phylogenetic analysis of SINA proteins in apple, *Arabidopsis* and rice to gain additional insight into the evolutionary relationships of apple SINA proteins (**Figure 1**). The 22 SINA proteins used in the analysis were divided into two groups in these three species. Groups I contained five MdSINA proteins (MdSINA1, MdSINA2, MdSINA5, MdSINA7, and MdSINA8), and Group II contained six proteins (MdSINA3, MdSINA4, MdSINA6, MdSINA9, MdSINA10, and MdSINA11). We noticed that all genes except MdSINA5 appeared in pairs based on the phylogenetic tree of the apple SINA family, implying possible gene duplication have occurred during the evolution of the SINA gene family. Subsequently, we used MCScanX to detect gene duplication events in MdSINAs in the apple genome. Five pairs, *MdSINA1/7*, *MdSINA2/8*, *MdSINA3/6*, *MdSINA4/9*, and *MdSINA10/11*, were localized in duplicated genomic regions (**Figure 2A**). And those genes which are flanked the MdSINA genes are also present in the syntenic blocks (data not shown), indicating that segmental duplication events occurred. Moreover, 11 apple SINA genes distributed across eight chromosomes, including 1, 2, 3, 6, 7, 11, 12, 14 (**Figure 2B**), which have highlighted strong collinearity between large segments of chromosomes 3 and 11, and between shorter segments of chromosomes 1 and 7, 2 and 7, 12 and 14 (Velasco et al., 2010).

### Gene Structure and Multiple Sequences Alignment Analyses of *MdSINA* Genes

**Figure 3:**
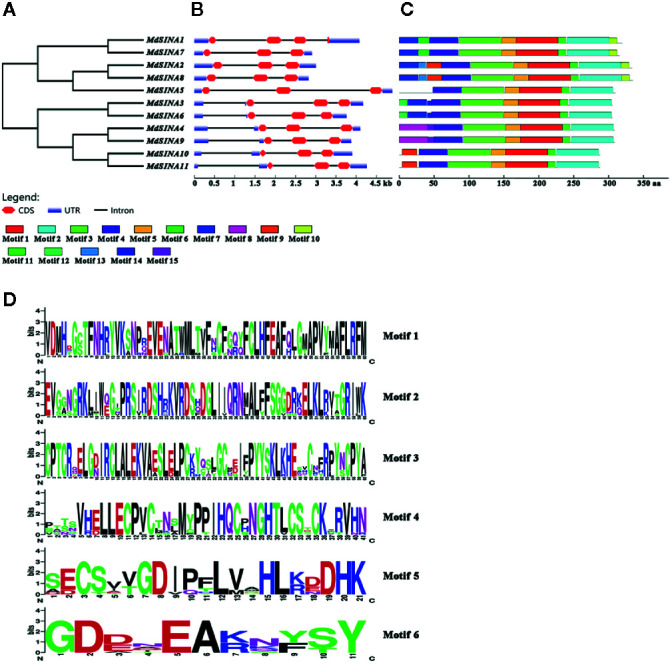
Gene characterization and structural analysis of *MdSINA*. **(A)** Phylogenetic tree of *MdSINA* genes. **(B)** Gene structure analysis of *MdSINA* genes was performed by GSDS. Red blocks and black lines represent exons and introns, respectively. **(C)** Conserved motifs of MdSINAs were detected by MEME. **(D)** Sequence logos of the conserved motifs. The degree of conservation was represented by the overall height of each position, while the height of each letter was proportional to its frequency.

The exon-intron structure of the *MdSINA* genes was examined using the online server GSDS to provide more insight into the evolution of these genes (**Figures 3A, B**). Gene structures were highly similar within each of the two group in terms of exon number. Furthermore, a similar exon-intron structure was observed within the same group of *MdSINA*. For example, Group I genes contained two introns and three exons except for *MdSINA1*. However, Group II genes possessed three introns and three exons. Although the protein sequence similarity between MdSINA1 and MdSINA7 was over 95% (data not shown), the structures of their associated gene differed. These differences implied that these two genes possessed divergent functions during their evolution.

The conserved motifs of apple SINA proteins were confirmed by MEME, and 15 distinct motifs were identified (**Figure 3C**). Motifs 1–6 were observed in all MdSINA proteins and contained conserved RING and SINA domains. Motif 4 was identified as the RING domain, while motifs 1, 2, 3, 5, and 6 were components of the SINA domain. Most members of Group I had motif 10 at the C‐terminus, while all Group II members lacked this motif. Five gene pairs had the similar, unique motif structure. For example, motifs 11 and13 were the unique motifs in *MdSINA1/7* and *MdSINA2/8*, respectively. The conservation of *SINA* genes during evolution was substantiated by the distribution of motifs. The uniqueness of the motif distribution in different gene pairs reflects the conservation and differentiation of *SINA* genes function during evolution. Moreover, the consensus sequences of motifs is stacked, suggested that all *MdSINA* genes shared highly conserved or completely conserved sites (**Figure 3D**).

**Figure 4:**
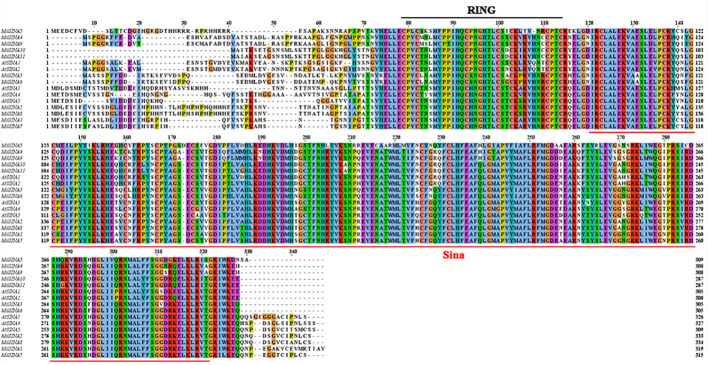
Multiple sequences alignment of SINA Proteins in apple and *Arabidopsis*.

We performed multiple sequences alignment to detect the conserved domain of MdSINA proteins (**Figure 4**). In the BLASTP analysis, the highest percentage of amino acid sequence identity obtained was between pairs of *MdSINA* genes, and these genes were also closely grouped in the phylogenetic tree. Our results suggest that an N‐terminal C3HC4-type (Cys-X2-Cys-X-Cys-X-His-X-Cys-X2-Cys-X-Cys-X2-Cys) RING domain and a C-terminal SINA domain occurred in all members. The RING domain is generally composed of eight conserved cysteine (Cys) and histidine (His) residues, and these eight conserved amino acid residues can bind two zinc ions to form a zinc-finger structure.

### Expression Patterns of *MdSINAs* in Different Tissues

**Figure 5:**
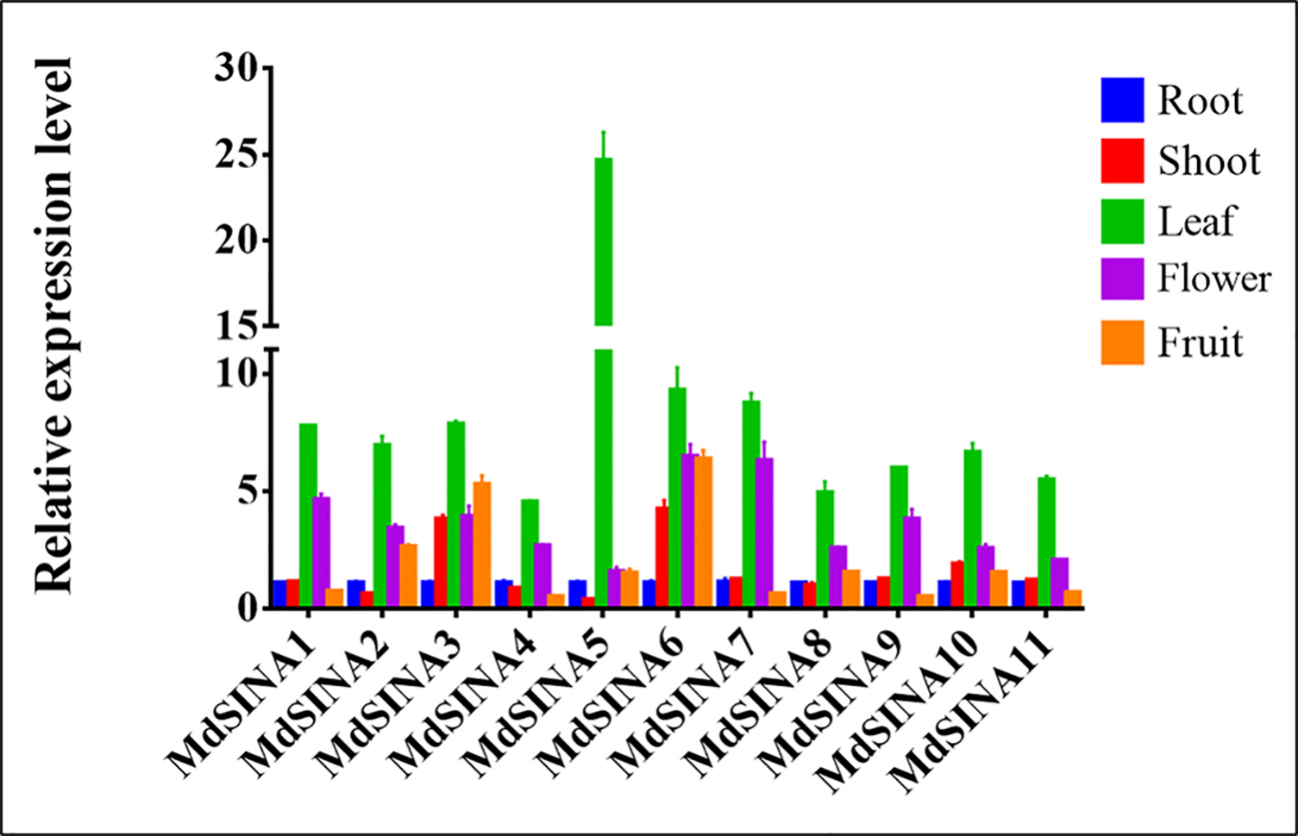
Expression patterns of *MdSINA* genes in different tissue. Transcripts of the 11 *MdSINA* genes in different tissues were determined by quantitative real-time PCR. Transcript values of the *MdSINA* genes determined in roots were set to 1. The apple *Actin 18s* gene was used as the reference transcript. Values are means ± SE of three biological replicates.

SINAs, from both animals and plants, have been found to play vital roles in different physiological processes. In order to further investigate the roles of *MdSINAs* in apple growth and development, testing the expression level of *MdSINAs* in different tissues is necessary. We performed qRT-PCR analysis to examine the expression levels of *MdSINAs* in a variety of tissues, including root, stem, leaf, flower, and fruit. The five gene pairs exhibited similar spatial expression patterns (**Figure 5**). *MdSINA1/7, MdSINA2/8, MdSINA4/9*, and *MdSINA10/11* showed relatively high expression levels in leaves and flowers, suggesting that these genes may be involved in leaf and flower development. In addition, *MdSINA3*/*6* showed similar expression patterns with higher expression levels in stems, leaves, flowers, and fruit, indicating that they may play an important role in the growth and development of apples. *MdSINA5* was only highly expressed in leaves. Overall the distinct spatial expression patterns imply that these genes play diverse roles in apple growth and development.

### Expression Patterns of *MdSINAs* in Response to ABA and Nutrient Starvation

**Figure 6:**
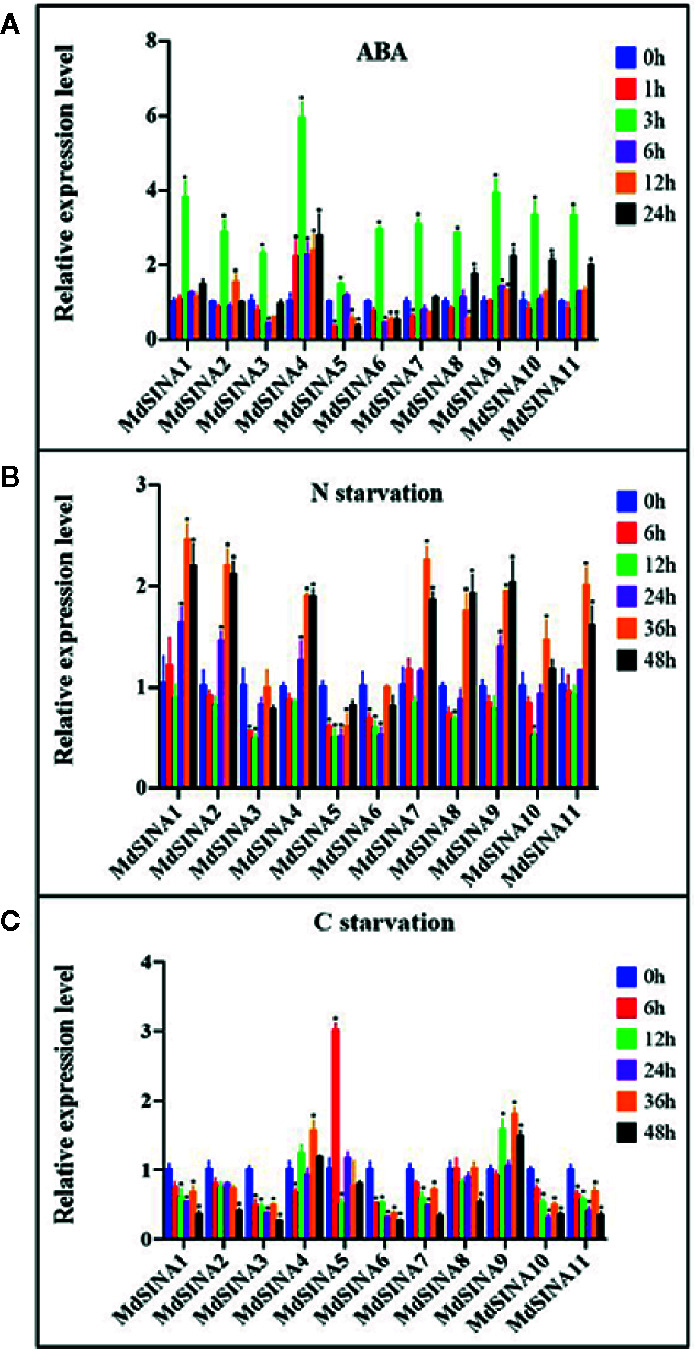
Expression profiles of *MdSINA*s in response to ABA **(A)**, nitrate-starvation **(B)**, and carbon-starvation **(C).** Transcript values of *MdSINAs* determined at 0 h were set to 1. The apple *Actin 18s* gene was used as the reference transcript. Values are means ± SE of three biological replicates. Significant difference are denoted by an asterisk (*P < 0.05).

A previous study has shown that *SINAT2* promotes drought tolerance in an ABA-dependent manner in *Arabidopsis* (Bao et al., 2014). To explore the potential response to ABA, the expression levels of *MdSINAs* were detected under ABA (100 μM) treatment. The expression levels of *MdSINAs* were highest at 3 h (approximately3–6-fold), and then returned to normal levels (**Figure 6A**). In addition, some of these genes had a higher expression level at 24 h, implying that MdSINAs were involved in ABA response and may act as important regulators of drought resistance as has been described previously.

Recently, studies have shown that SINAT1/2/6 are involved in starvation-induced autophagy in *Arabidopsis* (Nolan et al., 2017; Qi et al., 2017; Qi et al., 2020). To further characterize the potential roles of *MdSINA* genes in nutrient starvation, the expression levels of *MdSINAs* were examined under carbon- and nitrogen-starvation conditions. Under carbon starvation, *MdSINA4/9* were slightly up-regulated, and *MdSINA5* was significantly up-regulated (**Figure 6B**). Under nitrate-starvation conditions, the expression levels of *MdSINA3/5/6* changed little; however, the expression of other *MdSINAs* was markedly up-regulated (**Figure 6C**). Thus, *MdSINAs* may be involved in the process of autophagy and function in different stress responses.

### Interactions Among MdSINA Proteins

**Figure 7:**
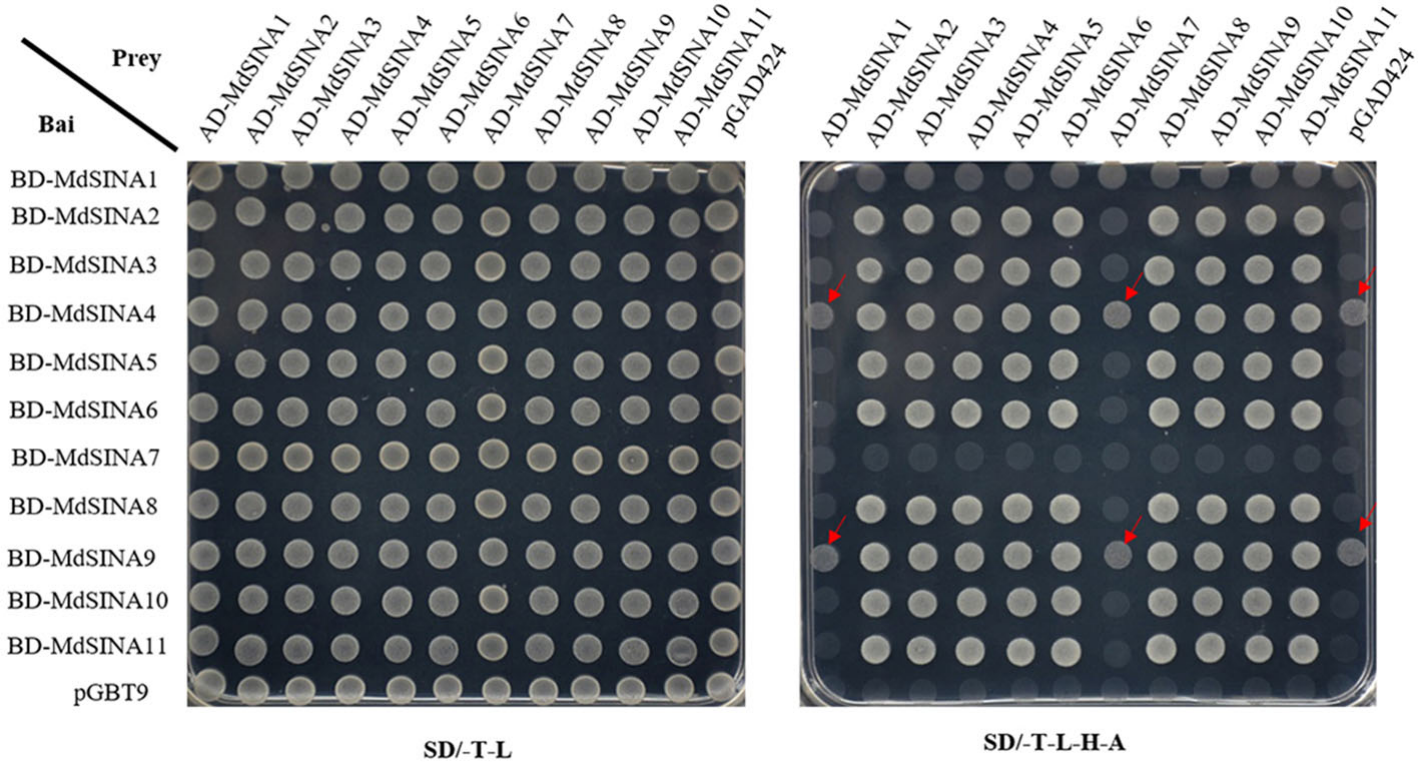
Y2H assay to test pair-wise interactions among MdSINA proteins. The yeast vector pGAD424 and pGBT9 were used as negative controls. The arrow indicates that BD-SINA4/9 showed weak autonomous activation.

Previous studies have suggested that SINA proteins can form both homodimers and heterodimers to regulate their own stability *via* the proteasome degradation pathway and perform different biological functions (Hu and Fearon, 1999; Xie et al., 2002; Depaux et al., 2006; Wang et al., 2018). The homo- and hetero-dimerization of MdSINA proteins was confirmed by Y2H experiments. First, the full-length cDNA of each *MdSINA* gene was cloned into the bait vector pGBT9 and the prey vector pGAD424. The fusion plasmids were then co-transformed into yeast cells (the “Y2H Gold” strain) grown on selection medium. All MdSINA proteins except MdSINA1 and MdSINA7 can interact with each other to form homodimers and heterodimers, implying that MdSINA1/7 might function as a single E3 ligase (**Figure 7**). Specifically, MdSINA4/9-BD show weak autonomous activation. However, MdSINA4/9-AD can interact with other MdSINAs proteins except for MdSINA1 and MdSINA7. Thus MdSINAs can form homodimers and heterodimers.

### Subcellular Localization of MdSINA2 Proteins

The predicted subcellular localization of MdSINA proteins is shown in **Table 1**, and MdSINA2 was selected for verification. Considering that ubiquitination can occur in the cytoplasm and nucleus to regulate the stability of cytoplasmic and nuclear proteins, the localization of ubiquitin ligase must localize in the cytoplasm, nucleus, or both (Xie et al., 2002; Heck et al., 2010; Yoo et al., 2013; Wang et al., 2018). To determine the subcellular localization of MdSINA2, we constructed the fusion plasmid *35S::MdSINA2-GFP* and injected it into the epidermal cells of *N. benthamiana* leaves *via Agrobacterium*-mediated transient expression. After 48 h, we observed the GFP signal under confocal laser scanning microscopy. The fluorescent GFP signal was distributed throughout the cells or only in the nucleus of the epidermal cells of *N. benthamiana* leaves, which were injected with either *35S::GFP* or *35S::GFP-MdSINA2*, respectively (**Figure S1**). Thus MdSINA2 was localized in the nucleus, which was consistent with our initial prediction.

### Light Signaling Mediates MdSINA2 Stability in a RING Domain-Dependent Manner

**Figure 8:**
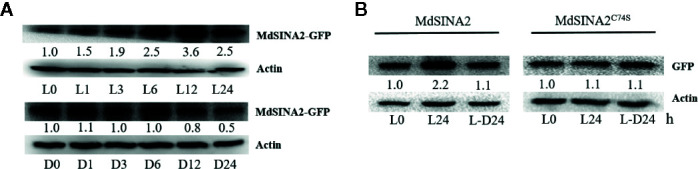
Light mediates the stability of MdSINA2. **(A)** MdSINA2 protein increased under light and decreased under dark condition. **(B)** MdSINA2^C74S^ protein was more stable than MdSINA2 both under light and darkness. Two-week-old *35S::MdSINA2* and *35S::MdSINA2^C74S^* apple calli were grown under continuous light (L) or dark (D) for the indicated time. L, continuous light; L-D, the continuous light-grown calli were transferred to the dark. MdSINA2-GFP was detected with anti-GFP antibodies. Actin was used for the loading control. The protein levels of MdSINA2-GFP determined at 0 h were set to 1. The assays were conducted in three biological replicates.

To gain further insight into the roles of *MdSINA2*, we cloned the full-length cDNA of *MdSINA2* into plant expression vector with GFP label and transformed it into apple calli (**Figure S2**). SINA proteins are E3 ubiquitin ligases that have important functions in plants and animals, and understanding the factors that affect the stability of MdSINA proteins is important. In *A. thaliana*, levels of SINA proteins increase under light and decrease in darkness. However, SINAT5 protein without a RING domain, which represents the Columbia ecotype (Col-0), is insensitive to dark and light treatment (Yang et al., 2017). Therefore, we determined whether the effect of light on the stability of the SINA protein in apple was the same as that observed in *Arabidopsis*. Two-week-old *MdSINA2* transgenic calli were grown on MS medium for 24 h under continuous light or dark conditions. We then transferred them to the dark or light for 1, 3, 6, 12, or 24 h. The protein level of MdSINA2 dramatically increased under light but decreased under dark conditions (**Figure 8A**).

Disruption of the ubiquitination site of the SINA protein is known to result in the loss of self-ubiquitination activity (Xie et al., 2002; Wang et al., 2018). To test whether the degradation of MdSINA2 protein depends on the RING domain, we obtained a mutant protein, Cys74→Ser (C74S), in which the RING domain was disrupted following the methods of a previous study (Xie et al., 2002). Although the MdSINA2 protein level in MdSINA2-GFP calli increased and decreased under light and darkness, respectively, there were no other noticeable differences between MdSINA2^C74S^-GFP calli (**Figure 8B**). Thus, the stability of light-mediated MdSINA2 protein is dependent on its RING domain.

### MdSINA2 Improves Sensitivity to ABA Treatment in Transgenic Apple Calli and Seed Germination of *Arabidopsis*

**Figure 9:**
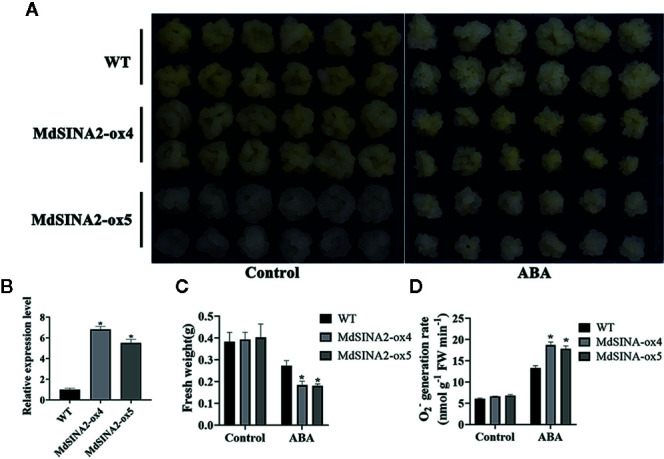
Overexpression of MdSINA2 results in sensitivity to ABA treatment in transgenic apple calli. **(A)** Phenotype of transgenic and WT calli grown in MS medium supplemented with 100 μM ABA. **(B)** Transcripts analysis of *MdSINA2* in transgenic and WT calli. **(C)** Fresh weight statistics of transgenic and WT calli in **(A)**. **(D)** O_2_^-^ generation rates of transgenic and WT calli in (A). Values are means ± SE of three biological replicates. Significant differences between groups are indicated by an asterisk (*P < 0.05).

*MdSINA2* transcripts were significantly induced by ABA treatment. To evaluate the role of *MdSINA2* in the ABA response, 2-week-old *MdSINA2* transgenic calli and WT were grown on MS medium with 100 µM ABA. *MdSINA2-OX* calli grew much slower than WT (**Figures 9A**, **B**). Moreover, *MdSINA2-OX* calli had lower fresh weight and accumulated more superoxide anions than WT (**Figures 9C, D**).

**Figure 10:**
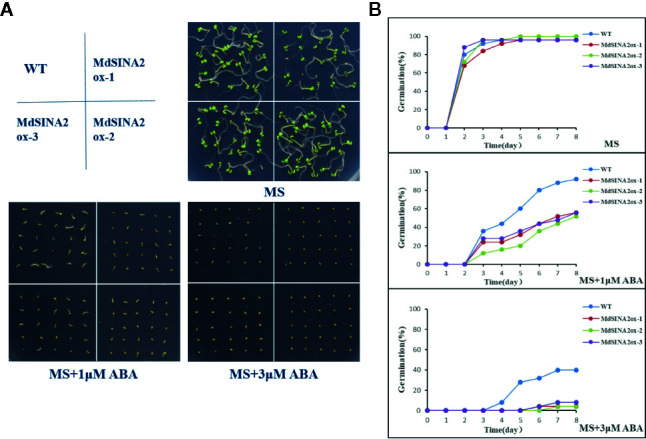
Overexpression of *MdSINA2* in *Arabidopsis* results in a lower germination rate under different ABA concentration treatments. **(A)** Seed germination of WT and *MdSINA2-OX Arabidopsis* on MS medium supplement with 1 or 3 μM ABA. **(B)** Seed germination rate of WT and *MdSINA2-OX Arabidopsis* within 8 days.

In addition, *MdSINA2* was successfully transformed into wild-type Arabidopsis (**Figure S2**). Three independent lines were selected for seed germination experiments. The transgenic *Arabidopsis* seeds were sown on MS medium supplemented with different concentrations of ABA. The germination rate was negatively correlated with ABA concentration (**Figure 10**). There was no significant difference in germination rates between WT and transgenic seeds on MS medium. However, the germination rate of transgenic seeds was significantly lower on MS medium supplemented with 1 μM or 3 μM ABA compared with WT. Moreover, the transgenic *Arabidopsis* seeds hardly germinated on medium with 3 μM ABA even after 8 days. Therefore, overexpression of *MdSINA2* results in an ABA-hypersensitive phenotype.

## Discussion

The function of SINA (Seven in absentia) homologous proteins have been extensively studied in animals, especially in the medical field, and these studies have revealed that SINA proteins are associated with resistance to various diseases such as cancer (Liu et al., 2008; Cai et al., 2015; Siswanto et al., 2018). Recently, an increasing number of studies investigating the function of SINA homologs have been conducted in plants (Zhang et al., 2019). SINA E3 ligase regulates plant growth and the response to different stresses *via* degradation of multiple targeted proteins. In this study, 11 *MdSINA* genes were identified in apple by genome-wide analysis (**Table 1**). *MdSINA* genes were found to be unevenly distributed across eight chromosomes through chromosomal localization. Five SINA homologous proteins are known in *A. thaliana* (SINAT1–5), 10 homologous proteins in *Populus trichocarpa*, six homologous proteins in *O. sativa*, at least six homologous proteins in *Zea mays*, and six homologous proteins in both *Medicago truncatula* and *Solanum lycopersicum* (Den Herder et al., 2008; Wang et al., 2008; Wang et al., 2018). However, the 11 *SINA* genes in apple may reflect an evolutionary expansion of the *SINA* family.

Multiple sequence alignments indicated that all MdSINA proteins contained two conserved domains: a RING domain and a SINA domain. Phylogenetic analysis was conducted to characterize the evolutionary relationships of *SINA* genes in apple, *Arabidopsis*, and rice (**Figure 1**). The results showed that SINA genes could be divided into two groups (Groups I and II) that differed in gene structure and motif composition between Group I and II. For instance, genes in the same group, such as *MdSINA2* and *MdSINA8* or *MdSINA4* and *MdSINA9*, exhibited similar exon-intron structures and motif compositions. The main mechanism driving the evolution of *SINA* genes appears to be gene duplication followed by divergence (Ohno et al., 1968; Flagel and Wendel, 2009). A previous study has shown multiple segmental duplication events in *Arabidopsis*, poplar, and rice (Wang et al., 2008). Our results suggest that segmental duplication events have also occurred in apple. Five gene pairs showing similar gene structures and protein sequences imply that these proteins show some degree of functional redundancy, such as MdSINA2/8.

A more comprehensive characterization of the *MdSINA* gene family is necessary given the high functional diversity of SINA homologs that has been documented in different species. Therefore, qRT-PCR analyses of the expression patterns of *MdSINA* genes were conducted. These 11 *MdSINA* genes were differentially regulated at the transcriptional level in different apple tissues. The transcript levels of most MdSINA family members were significantly higher in leaves and lower in roots (**Figure 5**), which is in contrast with the transcriptional patterns of these genes documented in tomato (Wang et al., 2018), implying that they might play an important role in leaf physiology in apple. Given that ABA signaling plays a vital role in regulating the response to different types of stress (Cutler et al., 2010), such as drought, cold, and salt, we analyzed the expression profiles of *MdSINA* genes under ABA treatment. The expression levels of *MdSINA* genes were altered under different degrees of ABA induction, suggesting that *MdSINAs* might participate in the stress response as a functional component of the ABA signaling pathway.

Furthermore, increasing evidence has demonstrated that autophagy can be induced by nutrient starvation and transport needless proteins or damaged organelles into vacuoles for degradation and circulation (Lee et al., 2010; Avin-Wittenberg et al., 2015; Chen et al., 2017; Islam et al., 2019). A large number of autophagy-related proteins (ATGs) have been documented in plants; these ATGs play an important role in regulating core autophagy mechanisms (Soto-Burgos et al., 2018; Zhuang et al., 2018). To date, a few studies have shown that SINA homologs are involved in the autophagy process in *Arabidopsis*, and *SINAT-RNAi* plants exhibit a starvation hypersensitivity phenotype. SINAT1/2 were negative regulators in autophagy process under nutrient starvation condition by mediating the ubiquitylation and degradation of ATG6/13. Here, we observed different expression patterns of *MdSINAs* under carbon- and nitrogen-starvation treatments (**Figures 6B, C**), implying that MdSINAs may play different functions in different periods and under different starvation treatments.

The stability and activity of SINA ligases *in vivo* depend on their dimerization (Hu and Fearon, 1999; Polekhina et al., 2002; Depaux et al., 2006). Hence, we detected the dimerization of MdSINA proteins. The SINA proteins in apple are surprisingly different from those in tomato and *M. truncatula* (Den Herder et al., 2008; Wang et al., 2018), all of which can interact with each other. We found that MdSINA1/7 neither formed homodimers nor heterodimers with any of the other MdSINA proteins. Thus, MdSINA proteins may function *via* the formation of homodimers and heterodimers or in another manner.

SINA ubiquitin ligases have a wide range of functions in plants and animals. They can regulate the stability of many important proteins in plants and animals. Previous studies have shown that the ABA response factor can regulate the transcription level of SINA2, but it remains unclear how the protein levels of SINATs are regulated (Bao et al., 2014). The identification of key factors regulating the stability of SINA proteins could enhance our understanding of the function of SINA proteins. We found that the MdSINA2 degraded in the dark, and it became insensitive to light after its RING domain had been destroyed. Based on this result and those of previous studies (Qi et al., 2017; Qi et al., 2020), we speculate that the degradation of MdSINA2 in the dark may stem from the lack of energy available under dark conditions.

ABA plays an important biological role in stomatal closure, stress resistance, seed germination, and senescence (Finkelstein et al., 2002; Assmann, 2003; Zhu, 2016; Sussmilch et al., 2017). The observation that *MdSINA2* is significantly induced by ABA (**Figure 5**) and is highly expressed in leaves suggests that MdSINA2 may be a functional component of ABA signaling and may be involved in the tolerance of different types of stress by regulating leaf development. Indeed, when we compared the germination rate of wild type (Col-0) and 35S::MdSINA2 on MS media supplemented with different concentrations of ABA, significant differences were observed in seed germination (**Figure 10**). Our results differed from those documented for SINA2 in transgenic *Arabidopsis* (Bao et al., 2014) under different ABA concentration treatments, where no significant differences were observed among wild-type and experimental groups. Although the specific mechanism of MdSINA2 in the responses to ABA and different types of stress remains unclear, our results suggest that altering MdSINA2 expression can significantly improve sensitivity to ABA in transgenic calli and *Arabidopsis*. Additional screening of possible targets with which MdSINA2 could interact in ABA signaling pathways will help elucidate the role that it plays in ABA signaling.

In conclusion, 11 *MdSINA* genes were identified in the apple genome. Bioinformatics analyses and analyses of gene expression were conducted to characterize their roles of these genes in apple growth and development. The functional characterization suggested that apple SINA members may function by responding to ABA signaling. These findings provided new insight into the ability of plants to resist abiotic stress.

## Data Availability Statement

The datasets presented in this study can be found in online repositories. The names of the repository/repositories and accession number(s) can be found in the article/**Supplementary Material**.

## Author Contributions

H-LL and Y-JH conceived the experiments. H-LL, XW, X-LJ, and Z-WQ performed the research. H-LL, XW, and X-LJ analyzed the data. H-LL and XW wrote the manuscript. X-LJ and Y-JH revised the manuscript. All authors contributed to the article and approved the submitted version.

## Funding

The National Key Research and Development Program of China (2018YFD1000200), the National Natural Science Foundation of China (U1706202 and 31772288), the Ministry of Agriculture of China (CARS-27).

## Conflict of Interest

The authors declare that the research was conducted in the absence of any commercial or financial relationships that could be construed as a potential conflict of interest.
