# Supplementary material for: Genome-Wide Identification of Apple Ubiquitin SINA E3 Ligase and Functional Characterization of MdSINA2
Source: Front Plant Sci. 2020 Jul 24;11:1109. doi: 10.3389/fpls.2020.01109 (PMC7393226; doi:10.3389/fpls.2020.01109)
Supplement: Supplementary file 1 [file DataSheet_1.docx]

Supplementary Material

# Supplementary Figures and Tables

**Figure S1.** Subcellular localization of the MdSINA2 protein. Recombinant plasmid *35S::GFP-MdSINA2* and control *35S: :GFP* was transiently expressed in the epidermal cells of *N. benthamiana* leaves. Scale bar, 10 μm.

**Figure S2.** Figure S2. *MdSINA2* transgenic materials were identified. **(A)** *MdSINA2-OX* calli was verified by mobility of dNA in gel electrophoresis. The recombinant plasmid of PRI-MdSINA2 was used as a positive control. **(B)** *MdSINA2-OX* calli was verified by Western blotting. The protein extract of WT calli was used as a negative control. **(C)** *MdSINA2-OX Arabidopsis* was verified by mobility of dNA in gel electrophoresis. **(D)** DNA sequencing proved the successful acquisition of *MdSINA2^C74S^* transgenic calli.

**Table S1.** Primer sequences used in qRT-PCR analysis.

**Table S2.** Primer sequences used in Y2H assay.

**Figure S1.** Subcellular localization of the MdSINA2 protein. Recombinant plasmid *35S::GFP-MdSINA2* and control *35S::GFP* was transiently expressed in the epidermal cells of *N. benthamiana* leaves. Scale bar, 10 μm.


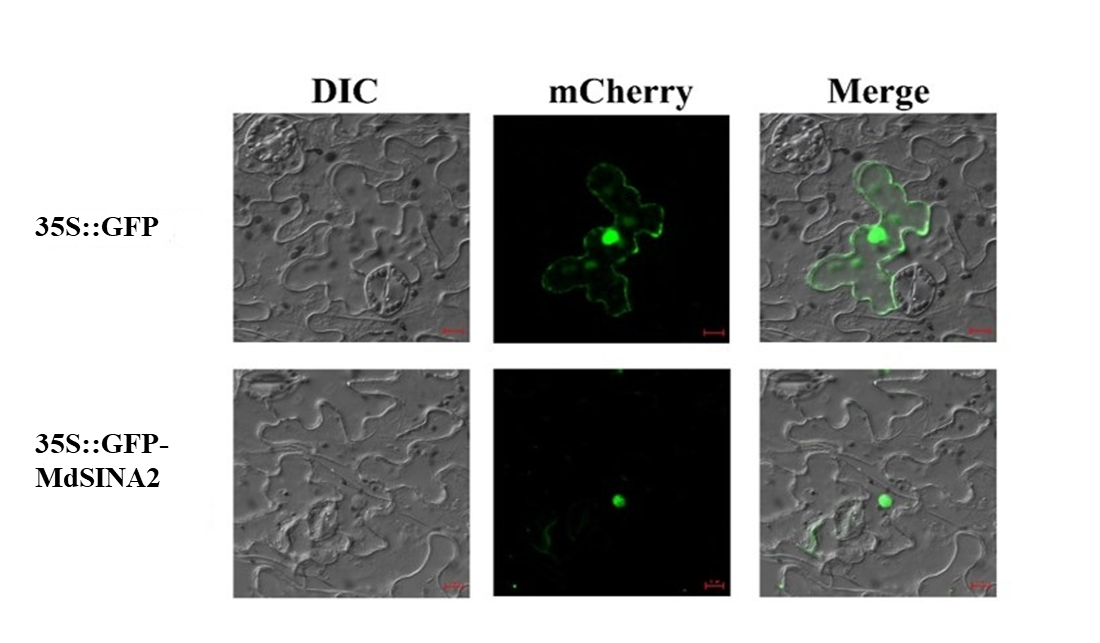


**Figure S2.** *MdSINA2* transgenic materials were identified. **(A)** *MdSINA2-OX* calli was verified by mobility of DNA in gel electrophoresis. The recombinant plasmid of *PRI-MdSINA2* was used as a positive control. (B) *MdSINA2-OX* calli was verified by Western blotting. The protein extract of WT calli was used as a negative control. (C) *MdSINA2-OX* *Arabidopsis* was verified by mobility of DNA in gel electrophoresis. (D) DNA sequencing proved the successful acquisition of *MdSINA2^C73S^* transgenic call.


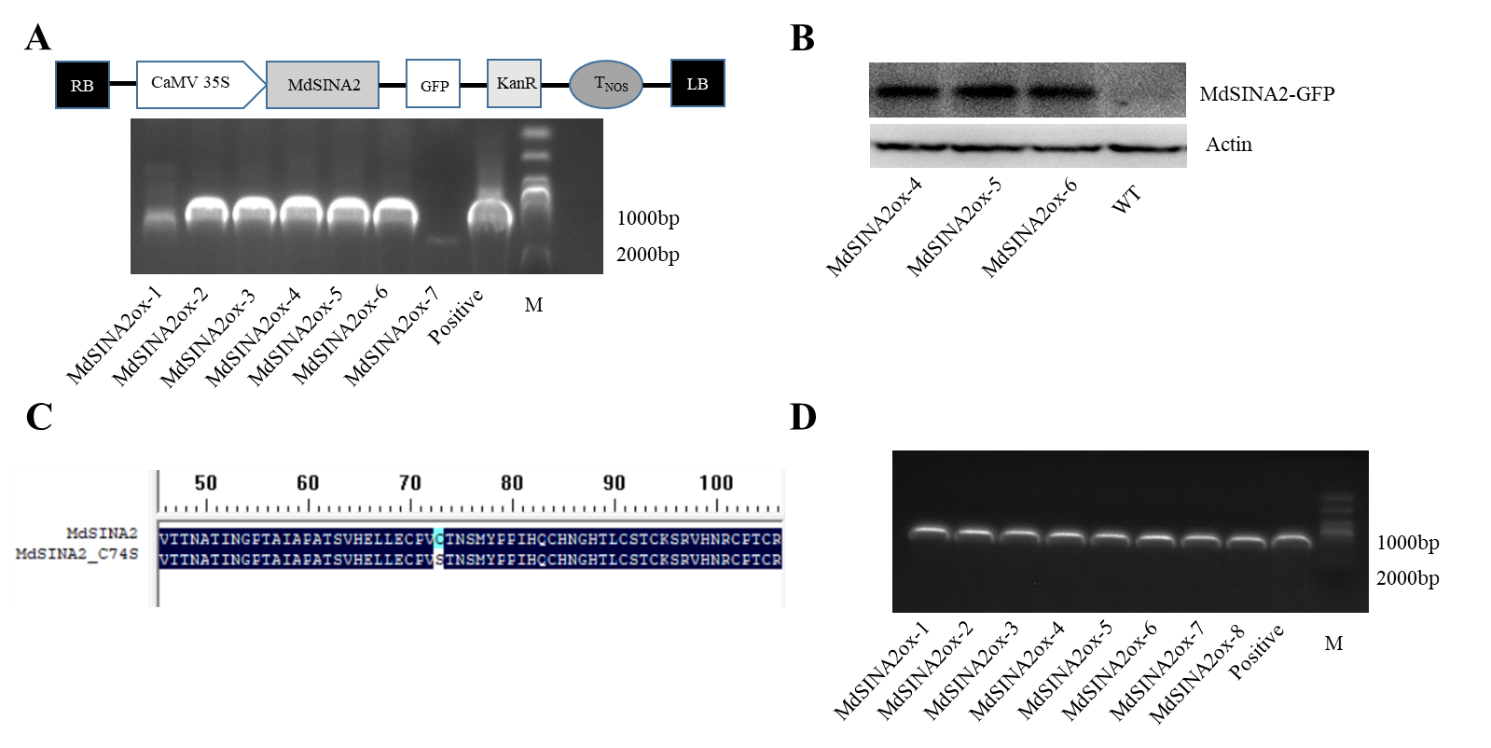


**Table S1.** Primer sequences used in qRT-PCR analysis.

| Primer name | sequence (5’ to 3’) |
| --- | --- |
| MdSINA1-F  MdSINA1-R  MdSINA2-F  MdSINA2-R  MdSINA3-F  MdSINA3-R  MdSINA4-F  MdSINA4-R  MdSINA5-F  MdSINA5-R  MdSINA6-F  MdSINA6-R  MdSINA7-F  MdSINA7-R  MdSINA8-F  MdSINA8-R  MdSINA9-F  MdSINA9-R  MdSINA10-F  MdSINA10-R  MdSINA11-F  MdSINA11-R | ATAATCGGTGCCCAACGTGT  CTGAGCACCCAAGAGAGCAA  CCTTTCCTGGTTGCCCATCT  TGTTAGCATCCATGTGGCGT  AACTGCCCCTATGCTGGTTC  TGAAAGTGCTGCCATTGTGC  ATGGGTGACGATGATGAGGC  TGACTGTCACGGACCTTTCG  TCGAGAAAAACTCGGCGACA  GGATCTCCATGCACCCCAAA  TGCCCTGTGTGCTTAAATGC  TGAACCCTTGGTTTGCAACC  ATGGCCGAAAGCTGATTTGG  GAAGAAAAGCGCCATGTTGC  AGCATGAATCGGTGTGCAAC  TGAATGTGCATCCTGTGTGC  TTGGCCGTCAGTTTTGCTTG  TGCTTCGCCTCATCATCATC  TGCACTTTGAGGCTTTCCAG  TTACGGCCATTTGCACCAAC  TTTGCACTTTGAGGCCTTCC  TTACGGCCATTTGCACCAAC |

**Table S2.** Primer sequences used in Y2H assay.

| Primer name | sequence (5’ to 3’) |
| --- | --- |
| MdSINA1-F  MdSINA1-R  MdSINA2-F  MdSINA2-R  MdSINA3-F  MdSINA3-R  MdSINA4-F  MdSINA4-R  MdSINA5-F  MdSINA5-R  MdSINA6-F  MdSINA6-R  MdSINA7-F  MdSINA7-R  MdSINA8-F  MdSINA8-R  MdSINA9-F  MdSINA9-R  MdSINA10-F  MdSINA10-R  MdSINA11-F  MdSINA11-R | ATGGAATCAGACATCATTGAAAGTCT  AACCGCAATCGTACGCATTACTTC  ATGGACTTGGAAAGCATCGAGT  GCTACACAGGTTTGGTATGCAC  ATGGCATCTAGTAGTCCATTTTTTGA  CTGTTCCTTCCATATCCTTCCGG  ATGTCTCCTGGAGGTCGCTTCTT  GTGTTCTTTCCATATACGTCCG  ATGGAGGAGGACTGTTTTGTTGATA  GGCACTGTTGTCCTTCCAAATC  ATGGCATCCAGTAGTCCATTTTTTG  CTGTTCCTTCCATATCCTTCCGG  ATGGAATCAGACATCATTGAAAGTT  GCTACAGAGAGGTATGCAGGTC  ATGGACTTGGAAAGCATCGAGTGT  GCTACACAGGTTTGCTATGCA  ATGTCTCCTGGAGGTCGCTTCTG  GTGTTCTTTCCATATACGGCCG  ATGGCAATCACAAAGTCAGAGACA  TTCTTCTTTCCATATACGGCCAG  ATGGCAATCGCTAAGTCGGA  TTCTTCTTTCCATATACGGCCAG |
